# A Post-Auricular Accessory Tragus

**DOI:** 10.7759/cureus.13645

**Published:** 2021-03-01

**Authors:** Michael Gebhard, Archana Shenoy, Julia Comer, Thomas Schrepfer

**Affiliations:** 1 Otolaryngology, University of Florida, Gainesville, USA; 2 Pathology and Laboratory Medicine, Nationwide Children’s Hospital, Columbus, USA

**Keywords:** congenital malformations, accessory tragus, embryology, pediatric otolaryngology, accessory auricle

## Abstract

Here, we document a rare and unique presentation of an accessory tragus (AT). A 3-year-old male presented with a 2-cm congenital post-auricular mass on his right side. Upon resection and histologic examination, the mass demonstrated histologic features consistent with an AT. To the best of our knowledge, this is the first reported case of an AT presenting posterior to the auricle.

## Introduction

An accessory tragus (AT) is a benign congenital malformation caused by an aberration of the first pharyngeal arch. This abnormality can occur as an isolated finding or with a syndrome, with the most common association being Goldenhar syndrome. AT can also be associated uncommonly with VACTERL syndrome, Wolf-Hirschhorn syndrome, and Townes-Brocks syndrome. Accessory tragi most often occur in the preauricular region anterior to the tragus but also commonly occur within the triangular area between the oral commissure and the anterior auricle [1]. Other rare localizations include the cheek, middle ear, glabella, and the lateral aspect of the neck [2,3]. The differential diagnosis of an AT includes ﻿acrochordon, auricular fistula, fibroma/fibroepithelial polyp, and epidermoid cyst [4]. ﻿Histological examination is required for a definitive diagnosis, and surgical excision is the most common form of management [5]. The histological criteria for the diagnosis of an AT requires the presence of a polypoid skin-covered lesion whose core includes abundant fibroadipose tissue and cutaneous adnexal structures including hair follicles with vellus hair, and in many cases, a central core of cartilage [6]. The presence of cartilage is not a requirement for the diagnosis, especially in a congenital mass that is in proximity to the ear or in the path of migration of the first branchial arch.

## Case presentation

**Figure 1:**
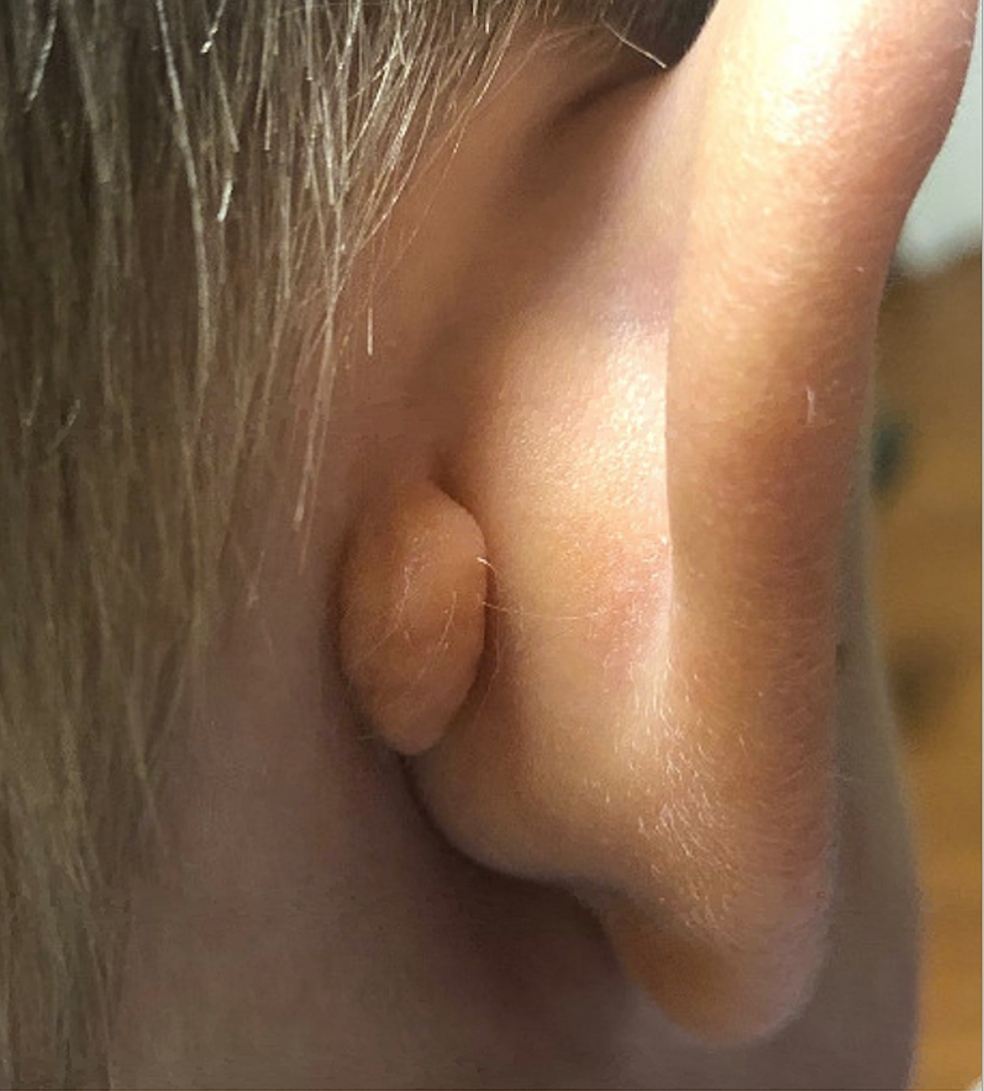
Pre-operative photograph of the post-auricular AT. AT, accessory tragus

A 3-year-old male was referred to a pediatric otolaryngologist by his pediatrician for a mass behind his right ear. The mass was noticed at birth and had been increasing in size. The mass was not painful, had no drainage, and the patient had no signs of hearing loss. The child had an uncomplicated pregnancy and a full-term delivery. The patient did not have any congenital syndrome. A complete review of systems was obtained and found to be negative. On physical examination, the pediatric otolaryngologist described the mass as a 2-cm fibroma behind the right ear that was soft and mobile (Figure 1). The mass was assessed to be a retro-auricular fibroma and elective excision was recommended.

**Figure 2:**
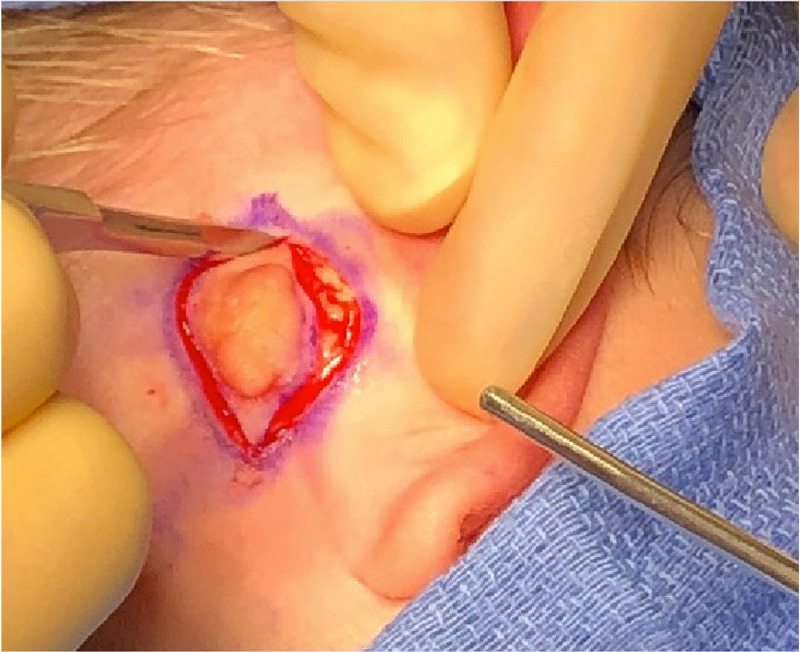
Intra-operative photograph of the AT. AT, accessory tragus

Six months later, the patient underwent elective excision. The post-auricular mass was excised under monitored anesthesia care, and the operative findings were consistent with the diagnosis of a fibroma (Figure 2).

**Figure 3:**
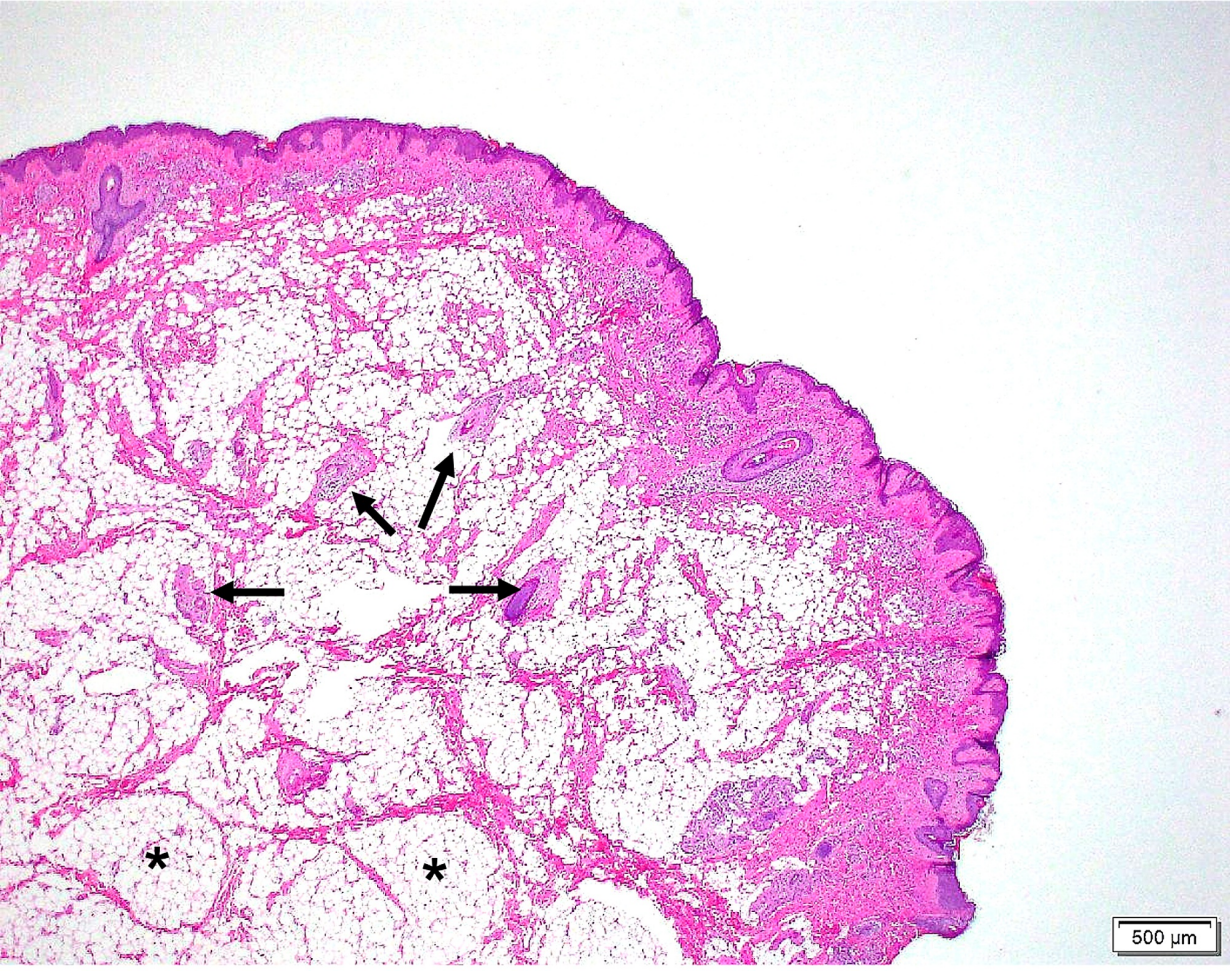
Hematoxylin and eosin-stained section (20× magnification) of the AT. Characteristic histologic features of an AT seen here include vellus hair follicles (arrows) and central fibroadipose tissue (*). AT, accessory tragus

The operative specimen was submitted to pathology. Histologic examination, as shown in Figure 3, demonstrated a polypoid structure with rugated epidermis on the surface. Underlying the epidermis, a complement of irregularly arranged vellus hair follicles with adnexal structures such as sebaceous glands and fibroadipose tissue were noted (Figure 3).

**Figure 4:**
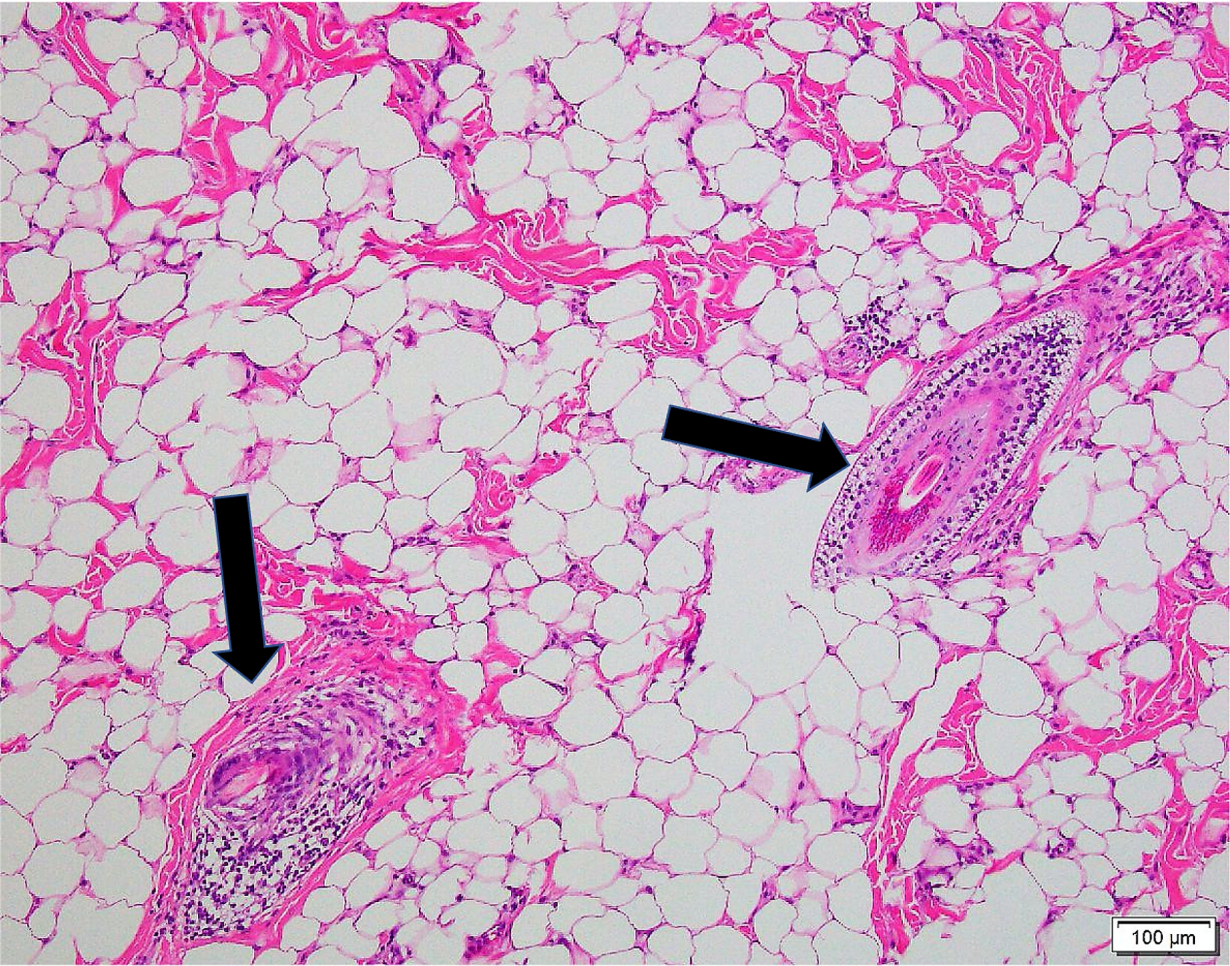
Hematoxylin and eosin-stained section (100× magnification) of the AT. Vellus hair follicles (arrows). AT, accessory tragus

Figure 4 shows the vellus hair follicles at greater magnification. Of note, no cartilage was identified in the histologic examination. Altogether, these findings were deemed consistent with an AT. The post-operative visit six weeks later showed a well-healed excision, and no further follow-up has occurred.

## Discussion

The posterior location of the AT found in this case is rare and unique. Embryologically, the anterior auricle develops from the first pharyngeal arch, which originates at the oral commissure [7]. This structure then migrates laterally and dorsally towards the final destination of the auricle. The first pharyngeal arch is composed of three hillocks that normally fuse together, and the failure of these hillocks to fuse is the accepted theory of how an AT develops. The most common locations of AT are along the migratory pathway between the oral commissure and the anterior auricle [5]. This case is unique because its location was lateral and posterior to the migratory pathway of the first pharyngeal arch. However, given the proximity of this AT to the tragus, it may be hypothesized that the distal most hillock was the one that failed to fuse.

Given the clinical examination findings, a fibroma was initially the presumed diagnosis. However, histologically, the complement of fibroadipose tissue and vellus hair follicles with cutaneous adnexal tissue was typical of an AT [5]. Of note, the lack of cartilage on histologic examination is not a requirement for the diagnosis of AT. Specimens that lack central cartilage can be mistaken for hair follicle nevi; however, an AT can be differentiated by the presence of prominent connective tissue network within the subcutaneous fat, which this case exhibits [6]. The presumed diagnosis clinically of this case was a fibroma, which is histologically characterized by a proliferation of hypocellular fibrovascular connective tissue that lacks cutaneous adnexal structures and vellus hair. The vellus hair follicles and central adipose tissue seen in Figure 3 excludes fibroma as a possible diagnosis in this mass. Additionally, the presence of this lesion at birth supports a developmental anomaly, which aligns with the diagnosis of an AT.

## Conclusions

To the best of our knowledge, this is the first reported case of an AT presenting posterior to the auricle. Accessory tragi most commonly present anterior to the tragus at various locations between the anterior auricle and the oral commissure or in the supraclavicular region. This case report, however, is unique because we observed an unusual presentation of an AT posterior to the auricle, which has not been described in the literature yet. The clinical findings of an AT can mimic other benign skin masses, but histological analysis can lead to a diagnosis. In the future, when patients present with congenital post-auricular masses, an AT may be considered in the differential diagnosis.
